# The cognitive basis of dyslexia in school‐aged children: A multiple case study in a transparent orthography

**DOI:** 10.1111/desc.13173

**Published:** 2021-09-09

**Authors:** Agnieszka Dębska, Magdalena Łuniewska, Julian Zubek, Katarzyna Chyl, Agnieszka Dynak, Gabriela Dzięgiel‐Fivet, Joanna Plewko, Katarzyna Jednoróg, Anna Grabowska

**Affiliations:** ^1^ Laboratory of Language Neurobiology Nencki Institute of Experimental Biology Polish Academy of Sciences Warsaw Poland; ^2^ Faculty of Psychology University of Warsaw Warsaw Poland; ^3^ Faculty of Psychology SWPS University of Social Sciences and Humanities Warsaw Poland

**Keywords:** developmental dyslexia, double deficit, dyslexia subtypes, multiple case study, phonological awareness, rapid automatized naming

## Abstract

This study focuses on the role of numerous cognitive skills such as phonological awareness (PA), rapid automatized naming (RAN), visual and selective attention, auditory skills, and implicit learning in developmental dyslexia. We examined the (co)existence of cognitive deficits in dyslexia and assessed cognitive skills’ predictive value for reading. First, we compared school‐aged children with severe reading impairment (*n* = 51) to typical readers (*n* = 71) to explore the individual patterns of deficits in dyslexia. Children with dyslexia, as a group, presented low PA and RAN scores, as well as limited implicit learning skills. However, we found no differences in the other domains. We found a phonological deficit in 51% and a RAN deficit in 26% of children with dyslexia. These deficits coexisted in 14% of the children. Deficits in other cognitive domains were uncommon and most often coexisted with phonological or RAN deficits. Despite having a severe reading impairment, 26% of children with dyslexia did not present any of the tested deficits. Second, in a group of children presenting a wide range of reading abilities (*N* = 211), we analysed the relationship between cognitive skills and reading level. PA and RAN were independently related to reading abilities. Other skills did not explain any additional variance. The impact of PA and RAN on reading skills differed. While RAN was a consistent predictor of reading, PA predicted reading abilities particularly well in average and good readers with a smaller impact in poorer readers.

## 1 INTRODUCTION

Since the first reports about children who struggle to read, thousands of papers have tried to uncover the sources of reading disorder. Researchers have attempted to connect low reading scores with deficits in various cognitive areas (see examples of such approaches: Alt et al., 2017; Lipowska et al., 2011; Manis et al., 1997; Ziegler et al., 2009).

While this research has provided evidence of the specific role of phonological processing in dyslexia, it also showed that a single cognitive deficit is not sufficient to explain the heterogeneity in the dyslexic population. It is now widely accepted that the etiology of dyslexia is multifactorial and best explained by various risk factors that increase the likelihood of fulfilling diagnostic criteria. According to the Multiple Deficit Model (McGrath et al., 2020; Pennginton et al., 2006), neurodevelopmental disorders are explained by various genetic and environmental risk factors that increase the possibility of developing the disorder. However, it is not clear how these factors sum‐up or interact in the case of dyslexia. Although evidence for clearly separable subtypes of dyslexia is missing, dyslexia is a heterogeneous disorder and individuals differ in underlying aetiologies. The different symptoms might at least partly be associated with different cognitive profiles.

Selection of deficits tested by researchers as a potential source of reading impairment is based on the major theories of developmental dyslexia. The most acknowledged is the phonological deficit theory (Ramus & Szenkovits, 2008; Snowling, 1995, 1998). Support for the phonological theory comes from evidence that dyslexic individuals perform particularly poorly on tasks requiring phonological awareness (PA), that is, conscious segmentation and manipulation of speech sounds such as finding rhymes or phoneme deletion. PA at preschool ages is a good predictor for later literacy skills for speakers of different languages (e.g., Lonigan et al., 2000) including Polish (Krasowicz‐Kupis, 2009). Intervention studies (Hatcher et al., 1994; Hulme et al., 2012, reviewed by: Melby‐Lervåg et al., 2012) show beneficial effects of early phonological training on further reading skills, which provides further evidence for a causal relation between the two skills (Hulme & Snowling, 2009). The double deficit theory (Bowers & Wolf, 1993; Wolf & Bowers, 1999, 2016) also posits that phonological deficits are central to dyslexia but adds deficient naming speed as an independent source of reading dysfunction. Their combined presence is then responsible for more profound reading dysfunction.

Difficulties with procedural learning or task automatization associated with cerebellar dysfunction may be linked to dyslexia (Nicolson & Fawcett, 2007). The cerebellum also plays a role in motor control including speech articulation, which, when dysfunctional, might lead to deficient phonological representations. However, Raberger and Wimmer (2003) showed that cerebellar deficit might be linked to co‐occurring disorders, such as ADHD, rather than dyslexia. Therefore, the causal relationship between cerebellar dysfunction and reading is much less clear than in case of phonological processing.

Dyslexia may be also related to sensory deficits and two theories have been forwarded to make this connection. One that is based on difficulties in auditory processing, like rhythm perception (rise time theory; Goswami, 2011) or tone perception (anchoring theory; Ahissar, 2007), which in turn may interrupt phonological processing. According to the rise time theory, the patterns of stressed and unstressed syllables in language may be processed by the same neural mechanisms that are used for processing patterns of strong and weak beats in music, at least in childhood. Hence individual differences in phonological processing in language should be related to individual differences in non‐linguistic musical tasks based on the patterns of the beat distribution. As for the anchoring theory, Ahissar et al. (2006) noticed that dyslexics’ detection of regularities in sound sequences is inefficient, which may also impair phonological processing.

**RESEARCH HIGHLIGHTS**

- This study tests the (co)existence of cognitive deficits in dyslexia in phonological awareness, rapid naming, visual and selective attention, auditory skills, and implicit learning.
- The most frequent deficits in Polish children with dyslexia included a phonological (51%) and a rapid naming deficit (26%), which coexisted in 14% of children.
- Despite severe reading impairment, 26% of children with dyslexia presented no deficits in measured cognitive abilities.
- RAN explains reading skills variability across the whole spectrum of reading ability; phonological skills explain variability best among average and good readers but not poor readers.

Another group of deficits encompasses problems in visual processing, such as visual attention (VA) span (Bosse et al., 2007) or selective attention (i.e., *attentional dyslexia*, for summary, see: Lukov et al., 2015). The VA span hypothesis postulates atypical development of reading skills because of non‐optimal grain size parsing mechanisms of orthographic inputs, for example, reduced quantity of information that can be processed at a glance simultaneously. Alternatively, the impact of attention deficit on dyslexia was linked to impairment in binding letters to words or difficulty in shifting attention during reading (Lukov et al., 2015).

The possible coexistence of several deficits in people with dyslexia has been considered and experimentally addressed in multiple case studies (Ramus et al., 2003; Sprenger‐Charolles et al., 2009; White et al., 2006). These studies showed that the most frequent deficit among both children and adults with dyslexia was a phonological deficit, in line with the phonological theory of dyslexia (Ramus & Szenkovits, 2008; Snowling, 1995; Snowling, 1998). Nevertheless, in several studies, the phonological deficit was present in only 50% of the sample, and there was a group of participants with dyslexia without any additional deficit (Reid et al., 2007; Sprenger‐Charolles et al., 2009; White et al., 2006, but see also Ramus et al., 2006). The phonological deficit was often found to coexist with other deficits (auditory, visual, or motor). It is also worth noting that rapid automatized naming (RAN) tasks were often included in phonological skills when opaque languages were studied (Ramus et al., 2003; White et al., 2006). Therefore, it is impossible to distinguish between the phonological and RAN deficits in some of the previous studies. However, this is not always the case, and a more recent study on French‐speaking children (Saksida et al., 2016) differentiated between phonological accuracy (measured with standard phonological tasks: phoneme deletion and spoonerism) and phonological speed (assessed with RAN tasks). This study showed that these two deficits affected a much higher number of children with dyslexia than visual deficits and that phonological and RAN deficits coexisted in the vast majority of participants (Saksida et al., 2016). Importantly, in a transparent (Polish) orthography, RAN deficit was at the same time the most frequent one among adults with dyslexia (Reid et al., 2007).

The previous multiple case studies involved relatively small groups (Ramus et al., 2003; Reid et al., 2007; White et al., 2006). The small samples not only limited the statistical power of the between‐group (Sassenberg & Ditrich, 2019; Szucs & Ioannidis, 2017) but particularly affected the method of finding deficits. Namely, a small control group cannot be representative for typical readers, and therefore the thresholds of deficits established based on the range of scores in such groups may not indicate real deficits. Additionally, some studies applied statistical procedures that resulted in boosting scores of the control group. In particular, these studies removed the lowest scores in the control group before calculating the ranges of typical scores (Ramus et al., 2003; Reid et al., 2007; White et al., 2006). Therefore, the chances of finding a deficit in participants with dyslexia were higher (as mean scores of typical readers were overestimated and standard deviations were underestimated). In fact, some of the deficits found could be false positives. The existence of false positives in the described studies is also suggested by a much higher number of subjects with dyslexia without any particular deficit found in a study that employed a bigger (*n* = 86) control group (Sprenger‐Charolles et al., 2009).

As most multiple case studies of dyslexia have been performed in individuals reading an opaque orthography such as English or French, their findings cannot be easily generalized to transparent languages (Landerl et al., 2013; Sprenger‐Charolles et al., 2011). Moreover, previous studies on the subtypes of dyslexia in transparent languages (e.g., Heim et al., 2008; Jednoróg et al., 2014) focused rather on comparison of distinguishable subgroups of children with dyslexia than on estimating the prevalence of the deficits.

We aimed to explore the presence and coexistence of cognitive deficits in children with severe developmental dyslexia (*n* = 51) in a transparent (Polish) orthography. We take into account previous methodological and statistical problems from earlier multiple case studies of dyslexia. We wanted to explore (1) which cognitive deficits are present in children with developmental dyslexia, (2) which of the deficits are the most common among children with dyslexia, (3) what combination of deficits is the most frequent, and (4) how the number and the severity of cognitive deficits experienced by a child is related to her reading level. None of these questions have been answered so far for a transparent orthography.

Given the strong evidence for RAN and phonological deficits in reading impairment, we expected that a phonological and a RAN deficit should be observed in a majority of children with dyslexia and that these deficits or their combination should have the most detrimental effects on reading ability. For other potential cognitive skills, predictions are less clear given the mixed experimental findings (e.g., Raberger & Wimmer, 2003; Saksida et al., 2016).

Secondly, treating dyslexia as the low end of a continuum including normal reading skills, we wanted to test which cognitive skills are the best predictors of reading outcome when we took into account a large group of children with various reading skills. To do that, we analysed the impact of cognitive skills on reading abilities in children with a wide range of reading skills (*N* = 211). We expected the reading abilities to be positively related to the PA skills and RAN, as well as to the other auditory (rhythm perception, tone comparison), visual (selective attention, VA span), and cerebellar (implicit learning) skills known from other studies to have an impact on reading ability.

## 2 METHOD

### 2.1 Participants

Participants (total *N* = 211) were school‐aged children (7–12 years old, M = 10.06, SD = 1.09) ranging from second to fifth grade and with an uninterrupted educational history. The sample included typical readers and children with developmental dyslexia (see below).

Participants were involved in a project on the cognitive heterogeneity of dyslexia approved by the Ethical Committee of the University of Social Sciences and Humanities and in accordance with the Declaration of Helsinki. They were recruited through schools (parental gatherings), the project website, or psychological–pedagogical counselling centres. Written consents were acquired from the parents of the participants and all children gave oral consent to participate in the study. Participants were Polish‐speaking monolinguals, right‐handed, and born at term. None of them had any history of neurological illnesses or brain damage and none had symptoms of ADHD or low IQ (below 85). Nonverbal IQ was assessed with the Wechsler Intelligence Scale for Children – Revised (Matczak et al., 2008). Socioeconomic status (SES) was measured based on the Barratt Simplified Measure of Social Status (Barratt, 2012). Familial history of dyslexia (FHD) was identified when a child had a first‐degree relative with a formal diagnosis of developmental dyslexia or at least one parent who scored more than 0.4 points in the Adult Reading History Questionnaire (Black et al., 2012; Lefly & Pennington, 2000; Łuniewska et al., 2019).

The participants were representative of the general population in Poland in terms of language status (all participants were Polish‐speaking monolinguals, while only 0.5% of children living in Poland spoke a mother tongue other than Polish according to the Census in 2011; Główny Urząd Statystyczny, 2013). Overall, the sample (*N* = 211) presented lower reading skills than the general population (mean standardized reading accuracy score in the sample was M = 4.93, SD = 2.15; whereas the values for the general population are M = 5.5 and SD = 2.0; *t*(210) = 3.83, *p* < 0.001; see Table S2.1). The limited reading level of the sample resulted from the overrepresentation of children with dyslexia. When children with a severe reading impairment (see the next section) were excluded, the remaining sample (*n* = 160) presented marginally better reading accuracy than the general population (M = 5.79, SD = 1.71; *t*(159) = 2.13, *p* = 0.035). However, the participants were not representative of the general population in terms of where they lived (mostly children from the urban area of Warsaw were included in the study) and parental education (the parents were more educated than in the general population).

### 2.2 Dyslexia diagnosis

We applied a standardized battery of tests used to diagnose developmental dyslexia (Bogdanowicz et al., 2009) to distinguish children with and without reading impairment from grade 3 onwards. Children selected as having a severe reading disorder (*n* = 51) presented both low reading accuracy (they scored below 16th percentile in a single‐word reading test) and low reading fluency (they scored below the 16th percentile in at least one out of two tests: pseudoword reading or reading with a lexical decision). We applied a conservative inclusion criterion of dyslexia, based on both reading accuracy and fluency, to increase the reliability of our group assessment and expected effect sizes (as recommended in Ramus et al., 2008). Spelling was not included as a diagnostic criterion, but 42 out of 51 children assigned to the dyslexia group also had a spelling impairment (i.e., scored below 16th percentile in a writing to dictation test). This is reasonable since Polish is a relatively transparent orthography with higher grapheme‐to‐phoneme regularity in reading than in spelling (Schüppert et al., 2017).

Children who scored above the cut‐off point (16th percentile) in all reading tasks were assigned to the typically reading group (*n* = 71). None of the control children were impaired in spelling. Children who scored low either in reading accuracy or reading fluency (but not in both; *n* = 81) and children attending the second grade who were too young to identify dyslexia (*n* = 8) were excluded from the analyses of dyslexia subtypes.

Selected groups of typical readers and children with dyslexia did not differ in age and FHD but differed in sex, nonverbal IQ, and SES (see: Table 1). Therefore, we used these demographic variables as covariates in all analyses. We decided to include FHD and age in the regression model because they could show significant effects even if their mean values do not differ between groups.

**TABLE 1:** Demographic differences between control group (*n* = 71) and children with dyslexia (*n* = 51)

Characteristic	Control group (*n* = 71)	Children with dyslexia (*n* = 51)	Test	*p*‐value	Cohen's *d* [95% CI^a^]
Age in years	10.06 (0.89)	10.06 (0.10)	*t*(120) = −0.03	*p* = 0.970	*d *= 0.01 [−0.36, 0.37]
Sex: M = males, F = females	38 M 33 F	34 M 17 F	^χ2^(1) = 4.80	*p* = 0.030	NA
Nonverbal IQ	118 (14.4)	112 (12.5)	*t*(118) = −2.09	*p* = 0.039	*d *= 0.39 [0.02, 0.75]
Familial history of dyslexia (±)	45− 26+	27− 24+	^χ^ *^2^* (1) = 1.33	*p *= 0.240	NA
Socioeconomic status	106 (19)	98 (23)	*t*(118) = −2.01	*p *= 0.047	*d *= 0.37 [0.01, 0.74]

Means and (standard deviations). All *p*‐values are Bonferroni‐adjusted.

^a^ CI = confidence interval.

### 2.3 Procedure

All tests were performed in two sessions, each lasting around 45 min. The sessions were run individually and took place either in a quiet room at school or in a testing room at the Nencki Institute of Experimental Biology. The standardized reading, phonology, selective attention, and rapid naming tasks were performed with a paper‐pencil method. The rhythm perception, tone comparison, VA span, and implicit learning tasks were designed as animated computer games for this study. All animated visual stimuli were presented on ASUS laptops (BU400A‐W3097X) with a 14‐in. screen. All auditory stimuli were presented binaurally through headphones. Cedrus keyboards RB‐540 (24 cm × 16 cm) were used for all computerized tests. The keyboards contained four active buttons (1.9 cm × 3.8 cm each) arranged in a cross shape. Depending on the task, the action buttons were limited to right and left, up and down, or all four were used in the task. A detailed description of all tools used in the study is available in Supplementary Material S1 and the available data on reliability of the tools is presented in Supplementary Material S2.

### 2.4 Data analysis plan

We divided our analyses into two parts to answer how cognitive skills are related to dyslexia and reading skills. First, we aimed to examine the (co)existence of deficits in the group of children with dyslexia (*n* = 51) based on their performance in the cognitive tasks (in reference to the control group without any reading deficit, *n* = 71). Second, we wanted to establish which cognitive skills are the strongest predictors of the reading level in the population of school‐aged children with a wide range of reading skills (*N* = 211). The whole sample included children with dyslexia, the control group, and other average readers who neither fulfil dyslexia nor control group criteria. All analyses were performed in the R programming language (R Core Team, 2020). Datasets and scripts are available in OSF at https://osf.io/mj32v/.

#### 2.4.1 Calculating cognitive skills factors

**FIGURE 1:**
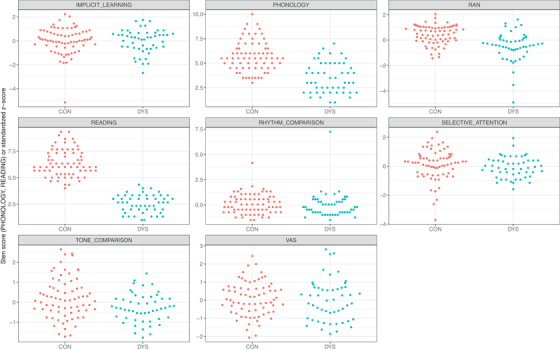
Distributions of individual performance values for each cognitive skill factor in the control group (CON) and in children with dyslexia (DYS)

We created variables that covered performance in all cognitive tasks and the reading skill variable. The READING variable was created by averaging normalized scores (stens) from the three reading tasks (single‐word reading, pseudoword reading, and reading with a lexical decision). The READING variable was an aggregated measure of reading accuracy and fluency. It was calculated independent of dyslexia diagnosis but was based on the same tasks. Correlations between the three variables ranged from 0.60 to 0.72. PHONOLOGY scores were established as averaged sten scores of the two phonological tasks: PA and phoneme deletion tasks (correlation 0.52). VA SPAN score was a sum of standardized scores from the partial and the global VA span tasks (Zoubrinetzky et al., 2014), transformed into z‐score afterward (correlation between task scores 0.29). In the case of other variables, that is, RAN, SELECTIVE ATTENTION, RHYTHM PERCEPTION, TONE COMPARISON, and IMPLICIT LEARNING we used final raw scores from the task transformed into z‐scores. We performed standardization based on the mean and SD from the whole group (*N* = 211) of children in all cases. In the case of RAN and TONE COMPARISON, the final score was reversed so that a higher score represented better performance (as in all other tasks). Distributions of values for all cognitive skill factors are presented in Figure 1. The descriptive statistics of the raw variable scores alongside reliability scores of the variables are shown in Supplementary Material S2 (Table S2.1).

#### 2.4.2 Differences between control and dyslexia groups

We tested differences between the control and dyslexia group with a logistic regression model predicting group membership based on the cognitive skills (with SES, FHD, nonverbal IQ, and age used as covariates). The final group size without missing data points was *N* = 118 (51 with dyslexia, 67 typical readers).

#### 2.4.3 Deficits in dyslexia

We aimed to identify one or more cognitive deficits in participants with dyslexia. For each cognitive skill, we chose a threshold for the deficit at the level of 1.65 SD (corresponding to the bottom 5th percentile) below the mean of the typically reading control group performance (cut‐off point used in similar studies, e.g., Ramus et al., 2003; Reid et al., 2007; White et al., 2006). In contrast with the previous studies, we did not exclude any participants from the control group who scored below −1.65 SD to reflect the variability of the typically reading group.

#### 2.4.4 Cognitive skills underlying reading

To perform analysis explaining reading level in the whole sample (regardless of the dyslexia diagnosis, *N* = 211), we selected standardized cognitive skills factors created previously. First, to study the relationship between different factors we ran a correlation analysis with a Holm‐Bonferroni correction for multiple comparisons on the whole sample (*N* = 211). Second, we built nested linear regression models with READING as a dependent variable and with other factors as independent variables. We entered age, nonverbal IQ, and SES as control variables in step 1 and added cognitive variables in step 2. The final group size without any missing data points in IQ and SES was *N* = 202.

#### 2.4.5 Controlling for age in cognitive skills factors

The READING and PHONOLOGY variables were based on age‐controlled sten scores. The scores were adjusted to the population norms (stens) on the basis of the educational stage of children. The norms calculated for the third grade were applied for all children attending the third grade (81 participants) and for children attending the first semester of the fourth grade (33 participants). The norms calculated for the fifth grade were applied for all children attending the fifth grade (58 participants) and for children attending the second semester of the fourth grade (32 participants). This procedure was consulted with the authors of the battery (Bogdanowicz et al., 2009). As the division into the two norming groups depended on the educational level of the child, not his/her age, the two groups partially overlapped in terms of age.

For factors other than READING and PHONOLOGY, we controlled for age using linear regression. For every factor, we fitted a linear regression model predicting the factor level based on age. Then, we used regression residuals as age‐controlled scores when calculating cognitive skill deficits in the dyslexia and control groups (participants’ age was partialled out from the raw score). In the regression analysis on the whole sample of participants, we used raw scores, since age was included independently as control in the full regression model.

## 3 RESULTS

### 3.1 Differences between control and dyslexia groups

A summary of logistic regression results is given in Table 2. Estimated coefficients may be interpreted as an increase of log‐odds (natural logarithm of P/(1‐P), P – probability) of belonging to the dyslexia group assuming an increase of predictor value of 1 SD. We identified differences in cognitive skills between children with dyslexia and typical readers for PHONOLOGY (log(OR) = −1.9, *p* < 0.001) and RAN (log(OR) = −1.9, *p* < 0.001).

**TABLE 2:** Coefficients of logistic regression predicting subject group (control or dyslexia) based on seven cognitive skills factors and four controls (*N* = 118)

Characteristic	log(OR)^a^	95% CI^a^	*p*‐value
FHD	0.05	−0.54, 0.62	0.874
Socioeconomic status	0.15	−0.47, 0.79	0.641
Age	0.51	−0.11, 1.20	0.118
Nonverbal IQ	−0.15	−0.81, 0.49	0.638
Phonology	−1.90	−2.80, −1.10	<0.001***
RAN	−1.90	−2.90, −1.10	<0.001***
Tone comparison	−0.29	−1.10, 0.46	0.458
VAS	−0.12	−0.71, 0.44	0.666
Selective attention	0.32	−0.32, 1.00	0.345
Rhythm comparison	−0.14	−0.63, 0.41	0.576
Implicit learning	−0.37	−1.00, 0.17	0.213

*p*‐values and confidence intervals from Wald test are given.

^a^ OR = odds ratio; CI = confidence interval.

***p < .001.

### 3.2 (Co)existence of deficits

**FIGURE 2:**
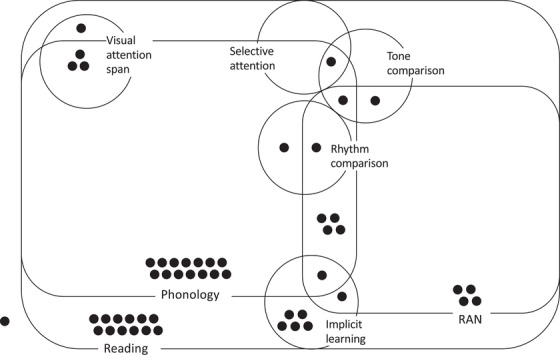
Distribution of the deficits in children with dyslexia

We grouped children with dyslexia by their cognitive deficits (see Figure 2). The two most common deficits within the dyslexia group were: PHONOLOGICAL (*n* = 26 out of 51; 51%) and RAN (*n* = 13; 26%). These deficits coexisted in seven subjects (14% of the group with dyslexia). However, 19 subjects showed neither of these two deficits, five of whom had a deficit in IMPLICIT LEARNING and one in VA SPAN. As the criteria used for deficit identification (−1.65 SD in the control group) differed from the criteria used for dyslexia diagnosis, one child assigned to the group with dyslexia presented no deficit in READING (z‐score in READING −1.61 SD relative to the control group). The detailed distribution of deficits (in both children with dyslexia and the control group) is provided in Tables S3.1–S3.4 in Supplementary Material S3. The distribution of deficits at thresholds −1.65 SD and −1SD are also provided.

### 3.3 Cognitive skills underlying reading

#### 3.3.1 Correlation analysis

Table 3 shows means and standard deviations of reading and cognitive variables, as well as correlations between all cognitive variables used in the study. Reading level was significantly and positively correlated with PHONOLOGY (*r*(211) = 0.49, *p *< 0.001), RAN (*r*(211) = 0.32, *p* < 0.001), and TONE COMPARISON (*r*(211) = 0.18, *p* = 0.009). The correlation matrix also showed weak intercorrelations between the cognitive variables.

**TABLE 3:** Pearson's correlations for cognitive skills variables (*N* = 211)

	1	2	3	4	5	6	7
1. Reading	—						
2. Phonology	0.49**	—					
3. Rapid automatized naming	0.32**	0.18*	—				
4. Tone comparison	0.16*	0.22*	0.10	—			
5. Rhythm comparison	0.05	0.15*	0.09	0.22*	—		
6. Selective attention	0.09	0.22*	0.30**	0.18*	0.13	—	
7. Visual attention (VA) span	−0.10	−0.04	0.02	0.05	0.07	−0.03	—
8. Nonverbal implicit learning	0.00	−0.06	−0.08	−0.03	−0.10	0.07	−0.04

*Note*: *p < 0.05. **p < 0.01. All *p*‐values are after Holm‐Bonferroni correction.

#### 3.3.2 Regression analyses

We performed a multi‐step linear regression analysis to investigate which variable could predict the variance in reading performance, corrected for the FHD, nonverbal IQ, age, and parental SES. Results are summarized in Table 4. In the first step, we used only control variables (no visible effects found). In the second step, controls and cognitive skills factors were used. The second model accounted for 29% of variance in reading (*F*(11, 190) = 8.45, *p* < 0.001, *R^2^* = 0.33, *R^2^_Adjusted_* = 0.29). Only PHONOLOGY and RAN levels were found to be significant predictors of READING (PHONOLOGY *β *= 0.43, RAN *β *= 0.30). In the third step, we included possible interactions between PHONOLOGY and RAN, PHONOLOGY and age, and RAN and age in the model, but they were not significant (*β *= 0.01, *p *= 0.888; *β *= 0.02, *p *= 0.751; *β *= 0.09, *p *= 0.154).

**TABLE 4:** Summary of linear regression models for variables predicting reading level (*N* = 202)

	Model 1			Model 2			Model 3		
	β	95% CI^a^	*p*‐value	β	95% CI^a^	*p*‐value	β	95% CI^a^	*p*‐value
FHD	−0.07	−0.21, 0.08	0.375	−0.06	−0.18, 0.06	0.328	−0.05	−0.18, 0.07	0.387
Socioeconomic status	0.05	−0.10, 0.19	0.538	−0.02	−0.15, 0.10	0.711	−0.01	−0.14, 0.12	0.852
Age	−0.06	−0.21, 0.08	0.378	−0.11	−0.25, 0.04	0.150	−0.11	−0.26, 0.04	0.137
Nonverbal IQ	0.11	−0.03, 0.26	0.116	0.01	−0.12, 0.14	0.897	−0.01	−0.14, 0.12	0.911
Phonology				0.43	0.30, 0.56	<0.001***	0.44	0.31, 0.57	<0.001***
RAN				0.30	0.17, 0.43	<0.001***	0.33	0.18, 0.47	<0.001***
Tone comparison				0.10	−0.03, 0.22	0.132	0.10	−0.03, 0.22	0.131
VAS				−0.06	−0.18, 0.07	0.386	−0.05	−0.18, 0.08	0.431
Selective attention				−0.07	−0.21, 0.07	0.299	−0.09	−0.23, 0.05	0.212
Rhythm comparison				−0.04	−0.16, 0.09	0.568	−0.04	−0.16, 0.09	0.577
Implicit learning				0.05	−0.07, 0.17	0.382	0.05	−0.08, 0.17	0.453
Phonology × Age interaction							0.02	−0.12, 0.16	0.751
RAN × Age interaction							0.09	−0.04, 0.23	0.154
Phonology × RAN interaction							0.01	−0.13, 0.15	0.888

Model 1 contains only control variables, Model 2 contains controls and cognitive skills factors, Model 3 includes possible interaction between phonology and RAN. Standardized coefficients with *p*‐values and confidence intervals from Wald test are given.

^a^ CI = confidence interval.

Model 1: R^2 ^= 0.029, adjusted R^2^ = 0.010, F(4, 197) = 1.496, *p*‐value = 0.205.

Model 2: R^2 ^= 0.329, adjusted R^2^ = 0.290, F(11, 190) = 8.449, *p*‐value < 0.001.

Model 3: R^2 ^= 0.337, adjusted R^2^ = 0.287, F(14, 187) = 6.787, *p*‐value < 0.001.

***p < .001.

We wanted to further investigate possible differences between the effects of PHONOLOGY and RAN on the reading level. We asked whether these effects are stable regardless of the range of READING values investigated. To answer this question, we sorted our data sample according to READING to obtain a sequence: s_1_, s_2_, …, s_n_. Then, we fitted regression models predicting READING using PHONOLOGY, RAN and controls as predictors on a sequence of subsamples of increasing size: {s_1_, …, s_20_}, {s_1_, …, s_21_}, …, {s_1_, …, s_n_} (the smallest subsample contained first 20 observations, the largest all 198 observations). From these models, we extracted standardized regression coefficients for PHONOLOGY and RAN. We performed this analysis with READING values sorted in ascending order and in descending order.

**FIGURE 3:**
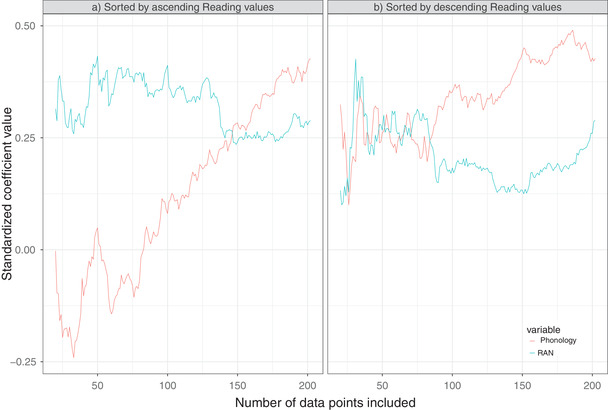
Sensitivity analysis of the regression coefficients as a function of the data sample. Linear regression model predicted reading level based on Phonology and RAN (with age, SES, FHD, and nonverbal IQ controlled for). The same model was fitted repeatedly using data samples of different sizes (sample size marked on the *x*‐axis), standardized coefficients of Phonology and RAN were extracted from the fitted model (standardized coefficients marked on the *y*‐axis). Samples were formed as subsets of the original sample increasing in size, while data points were sorted by Reading values (a) in ascending order, (b) in descending order. If the value of the standardized coefficient is similar for all sample sizes, it means that the predictor is equally good across the full range of Reading values

Results are presented in Figure 3. From this plot, we can see the value of the PHONOLOGY β coefficient when we consider only 100 children with the lowest reading level. The value of β for RAN oscillates around 0.25 regardless of the range of READING considered. For PHONOLOGY β grows with the sample size and reaches a maximum when the full sample is considered (Figure 3a and b). This suggests that PHONOLOGY is useful to explain large differences in READING across the whole scale. Furthermore, PHONOLOGY β is negative or close to zero when only low values of READING are considered (Figure 3a), which means that it does not explain variability among poor readers.

To better understand the results of the regression analyses, we repeated them for reading accuracy and reading fluency considered as separate factors. Very similar patterns of results were observed for both factors with PHONOLOGY and RAN as the main predictors. As with the combined factors, PHONOLOGY explained reading variability only in average and good readers and not in poor readers. We present detailed results in Supplementary Material S4.

## 4 DISCUSSION

We investigated which cognitive deficits are the most common in Polish children with dyslexia and which cognitive skills are the best predictors of reading skills. The most common deficits in children with dyslexia were phonological (51%) and RAN (26%), with 14% of children showing a double (RAN and phonological) deficit and 14% an implicit learning deficit. Other deficits were uncommon. Similarly, RAN and phonological skills were the only skills that differentiate between groups with dyslexia and good readers.

While searching for reading predictors in a large group of children with various reading skills, we found that RAN and phonological skills had the strongest impact on the reading scores. The impact was larger than for selective attention, implicit learning, tone comparison, rhythm comparison, and VA span. Results from the regression analysis, where both RAN and phonological skills explained a portion of the variance in the reading performance, indicated that these skills independently affected the reading scores. However, we did not find any interaction between RAN and phonological skills that would suggest an additive effect of both factors on the reading level. Our exploratory analysis of regression in which we tracked the changes in the impact of RAN and phonological skills on reading with increasing and decreasing reading performance showed that RAN has a consistent effect on reading scores across the whole spectrum of reading skills. On the other hand, although phonological skills explain reading performance in the whole group, they cannot explain the variability of reading skills in poor readers. In other words, below a certain reading performance level, the reading skills are no longer related to phonological skills. However, this result should be treated with caution because the phonological tasks used in our study (PA and phoneme deletion) were designed for assessment of phonological skills in the general population (and not for precise assessment of low phonological skills in the dyslexia population). Perhaps we observed no relation between reading skills and phonological skills in the lower end of the spectrum because of the limited range of low phonological scores.

Our results are in agreement with a previous multiple case study on English‐speaking children (White et al., 2006) where the majority of children with dyslexia (52%) had a phonological deficit. These results were interpreted in favour of the phonological deficit explanation of dyslexia. However, in that study the results from RAN tasks (object naming and digit naming) were included in the phonology factor. Another study on prevalence of cognitive deficits in French children with dyslexia showed that if RAN was separated from the phonological skills (it was labelled phonological speed), a phonological deficit was noted in 92% of children with dyslexia, a RAN deficit was observable in 85% of the children, and 79% of the children had a double phonological‐RAN deficit (Saksida et al., 2016). In our study, also treating RAN as a separate cognitive dimension influencing reading, we found only a weak correlation between RAN and phonological skills (see Swanson et al., 2003 for meta‐analysis) after correction for multiple comparisons (*r*(201) = 0.18, *p* < 0.05), which suggests that these two skills should not be treated as a joined factor. The association between these two skills might depend on the developmental stage or the transparency of the orthography (de Jong & van der Leij, 1999; Kirby et al., 2010; Landerl et al., 2013; Wimmer et al., 2000).

Our results emphasize the independent role of RAN and phonological skills in predicting the reading level. This is in line with the double‐deficit theory of dyslexia (Wolf & Bowers, 1999), where RAN and phonological skills are treated as two separate sources of reading difficulties and their combination leads to more severe impairment (Bowers & Wolf, 1993; Wolf & Bowers, 1999, 2016). Empirical evidence in favour of the double‐deficit was provided in studies on transparent (Boets et al., 2010; Papadopoulos et al., 2009; Torppa et al., 2012) and more opaque orthographies (Miller et al., 2006). Some typical readers in our sample presented isolated phonological (one child) or RAN (four children) deficits; however, there were no typical readers with a double deficit (Table S3.3). These results nicely correspond to another study on Polish children with dyslexia (*N* = 46, Jednoróg et al., 2014). In this study, three different clusters of children were distinguished: with phonological and magnocellular‐dorsal deficits, with RAN and auditory attention shifting, and with a double deficit. Even though auditory attention shifting and magnocellular‐dorsal deficit were not tested in this study and a clustering method differed from multiple‐case design, a similar pattern was found: children with dyslexia belong to three distinguishable clusters—with a phonological deficit, with a RAN deficit, or with a double deficit.

In our study, 14% of children with dyslexia showed an implicit learning deficit. This deficit was not accompanied by any other deficit in the case of five out of seven children. According to the cerebellar deficit hypothesis (Nicolson & Fawcett, 2007), alterations in the cerebellum may lead to impaired articulatory, phonological, and/or implicit learning skills, which in turn may contribute to the reading disorder. In our sample, only one child had coexisting phonological and implicit learning deficits. The clear distinction between phonological and implicit learning deficits may suggest that implicit learning is a skill that may explain a certain number of dyslexia cases independent of RAN and phonology.

Around 24% of children with dyslexia in our sample showed no apparent cognitive deficit in phonology, RAN, or implicit learning. Only a few of them present a single or coexistent deficit in other domains (attention, motor, auditory). The lack of deficits in almost a quarter of children with dyslexia might be surprising, as in the previous studies almost all participants with dyslexia presented at least one cognitive deficit (Ramus et al., 2003; Reid et al., 2007; White et al., 2006). However, in the study of Pennington et al. (2012), around 40% of dyslexia cases showed no deficit in PA or RAN (here: 37%). What's more, in the only multiple‐case study which used a substantially higher number of participants in the control group (*n* = 86), seven out of 15 poor readers (47%) did not present a phonological or RAN deficit (Sprenger‐Charolles et al., 2009). In other multiple case studies, the control and poor readers group sizes were similar and varied between 15 and 23 individuals (Ramus et al., 2003; Reid et al., 2007; White et al., 2006). The observed relation between the size of the control group and the number of children with dyslexia who did not present any particular deficit underlines the need for representative control groups in multiple case studies. Plausibly, in the previous studies, the criteria for deficits were too liberal. Although these studies applied the criterion of −1.65 SD below the scores of the control group much like we do in our work, the standard deviation could be underestimated because of the small sample size and exclusion of the lowest scores of the control group. This could result in false‐positive identification of deficits in the samples with dyslexia.

There are also other possible explanations for the lack of cognitive deficits in 24% of children with dyslexia. Perhaps the children were tested too late. Some deficits might be present early in development (Carroll et al., 2016) but may not be detectable at school‐age. Also, other cognitive deficits or environmental causes might interrupt learning processes that are not yet included in the main theories of dyslexia and were not controlled in the present study.

Finally, the criterion of −1.65 SD might be too strict to reveal the influence of all relevant deficits on dyslexia. Reading impairment may occur in the case of multiple weaker (instead of one stronger) deficits. To explore this possibility, we examined the patterns of deficits while applying a much more liberal criterion of deficit at −1SD (Tables S3.1–S3.4). We noted that weak phonological and RAN deficits did not lead to dyslexia when not accompanied by each other. Among children who scored below −1SD in PA alone there were 11 typical readers (39%) and 17 children with dyslexia (61%), and among children with a weak RAN deficit, there was an equal number of typical (*n* = 10) and impaired readers. However, among children who scored below −1SD in phonological and RAN skills at the same time, there were 95% of children with dyslexia (*n* = 18) and just one typical reader. This suggests that even if phonological and RAN deficits are weak but coexist, they may lead to dyslexia. This is plausibly the reason why we cannot detect more cases like the one mentioned above in the control group. The deficit cases in the control group are present as a consequence of the chosen statistical threshold (up to 76% of typical readers will present at least one score lower than −1SD when such a threshold is chosen). However, even if a child has a weak or even a strong deficit in one domain, it will not necessarily lead to reading problems. Nevertheless, the combination of phonological and RAN deficits seems to be damaging for reading skills in a transparent orthography. As for clinical implications, we cannot use the cognitive profiles to simply diagnose dyslexia instead of the reading level itself. A good practice would be to test phonological and RAN deficits. If they coexist, likely, a child will also have reading difficulties. Similarly, early dysfunction in RAN and phonology may lead to dyslexia.

Although our regression model was based on a wide set of cognitive skills, it explained only 30% of the variance in reading. This may seem surprisingly low. However, this is no different from some previous models which take into account cognitive skills like phonology or RAN (e.g., Compton et al., 2001; Landerl & Wimmer, 2008). More variance might be explained if something other than cognitive or socioeconomic factors were included in the models. Reading skills may be accounted for by the quality of education or even personality traits (Agler, Noguchi & Alfsen, 2019). On the other hand, there are studies with a larger group of participants where PA and/or RAN explained a higher portion of the variance in reading (e.g., Pennington et al., 2012, around 50%). So, including more participants in the study may lead to an increase in prediction value of our model.

In summary, our results showed that, in a transparent orthography, phonological and rapid naming factors are the most reliable predictors of the reading level in children with dyslexia and a group of children with various reading levels. Our results suggest additive influence of rapid naming and phonology on reading. Rapid naming appears to be the most stable predictor of reading, regardless of the reading level whereas phonology explains variability in average and good readers but does not explain variability in poor readers.

## CONFLICT OF INTEREST

None of the authors has a conflict of interest.

## Supporting information

Supporting information

## Data Availability

Thedata used for the current study is available from the Open Science Framework: https://osf.io/mj32v/
