# Supplementary material for: The cognitive basis of dyslexia in school‐aged children: A multiple case study in a transparent orthography
Source: Dev Sci. 2021 Sep 9;25(2):e13173. doi: 10.1111/desc.13173 (PMC9285470; doi:10.1111/desc.13173)

**Supplementary materials for the manuscript**

**The cognitive basis of dyslexia in school-aged children: a multiple case study in a transparent orthography**

# S1. Description of all cognitive and reading measures used in the study.

This supplementary material details the tools used for dyslexia diagnosis and for assessment of cognitive skills.

### Reading

Reading tests were a part of a standardized battery for dyslexia diagnosis in Poland [(Bogdanowicz et al., 2009)](https://www.zotero.org/google-docs/?zIepad). Reading accuracy was measured in a single-word reading test. Reading fluency was measured with pseudoword reading and reading with lexical decision tests. The single-word reading test for younger children (until the first semester of 4th grade) consisted of 50 words, and for older children (from the second semester of 4th grade) of 85 words. Words differed in the level of complexity and frequency of occurrence in Polish between the two versions. The two other reading tests had identical sets of items in the two versions. In the pseudoword reading test, the task was to accurately read as many items as possible from a list in 60 s (max. 70 items). In the reading with lexical decision test, the task was to cross out as many pseudowords as possible from the set of 78 items (50 real words and 28 pseudowords) in 60 s. The pseudowords used in these tests were pronounceable but had no close word neighbors. To control age and education level, the individual results in all tasks for every child were transformed into normalized (sten) scores based on the psychometric scale from the battery.

### Phonology

The phonological skills were assessed with two tests belonging to the normalized battery for dyslexia diagnosis (Bogdanowicz et al., 2009). The first, phonological awareness test, included seven subtasks on pseudoword stimuli: paronym analysis (25 items), syllable analysis (5 items), syllable synthesis (5 items), phoneme analysis (8 items), phoneme synthesis (8 items), and phonological memory (4 items). The outcome score was the sum of the raw scores in all subtasks. In the second test, the phoneme deletion test, participants were asked to delete a given phoneme from the heard word (e.g. say “banana” without “b”). The test consisted of 23 items. The final scores from both tests were transformed into a normalized (sten) score.

### Rapid automatized naming (RAN)

In the RAN test, children were asked to name two sets of either colors or objects as quickly as possible (48 items on each board, Polish version: Fecenec et al., 2013). The final score was the time (in seconds) needed to finish both boards. The final score was transformed into a normalized (sten) score.

### Selective attention

Visual selective attention test was part of the Intelligence and Development Scale (Jaworowska et al., 2012). Children were asked to identify as many items (ducks of given characteristics) as possible in the limited time of 15 seconds per row. Nine rows of target items and visually similar distractors were given. The final score was the number of correctly identified targets minus incorrectly identified items.

### Rhythm perception

In the computerized task of rhythm perception, children were asked to decide whether two short melodies (metrical arrangements) are the same or different. Training trials contained two pairs of melodies to compare with feedback provided. The design of the task was similar to the rise time task from Huss et al. (2011). Twenty-four experimental trials were created with sampled sounds from a vibraphone. In half of the trials, melodies were identical. Each melody lasted around 14 seconds and contained 2 to 4 tones repeated three times. The pitch of the musical notes was G (392 Hz). Tones were delivered every 500 ms (120 beats in one minute). 14 trials (7 same, 7 different) were presented with a 4/4 time signature, another 10 trials (5 same, 5 different) in 3/4 time with one more intense note in the sequence (by 5 dB). Different pairs of melodies were created by making the accented note longer in the second delivery (in 5 melodies for 166 ms, in another 7 for 100 ms). The accented tone may appear as a first (in the case of 14 trials), as a second (in 6 trials), or as a third (4 trials). Melodies were presented in a pseudo-random order with no more than three matched pairs in a row. The final score was the percentage of correctly judged pairs of melodies.

### Tone comparison

In the computerized tone comparison task, children were asked to decide which of the two tones sequentially delivered by animated birds had the higher pitch. The design of the task was similar to Ahissar’s et al., (2006) frequency discrimination task (version without the reference tone). In practice trials, children were given tones with a 1,000-Hz difference, with tone duration 50 ms and interstimulus interval 600 ms. Visual feedback was provided only in the practice trials. In the experimental trials, both tones changed from trial to trial and the experiment started from a 500 Hz difference between tones. The lower tone was randomly chosen from the interval 1,000-1,400 Hz. The higher tone was calculated in each trial based on the subject's performance. After two successive correct responses, the difference between tones was 40 Hz smaller, after an incorrect response 40 Hz higher. The task lasted for 70 trials or 16 changes in the direction of the frequency difference. Tone intensity was always 65 dB. The minimal difference between tones was never smaller than 5Hz. The final score was the tone discrimination threshold that was calculated as the mean of the frequency differences in the last seven reversals.

### Visual attention span (VAS)

Visual attention span abilities were assessed with two computerized tasks of global and partial symbol reporting (similar to Zoubrinetzky et al., 2014). For the global and partial symbol report task, quasi-random four symbol strings were built up from 8 symbols of similar visual complexity. The global report task included 16 four-symbol strings, preceded by five training trails with feedback provided. The strings contained no repeated symbols. The symbols were presented in white on blue background (see Figure 1A). Each symbol was used eight times and appeared twice in each position. At the beginning of each trial, a blank screen was presented for 1000 ms, followed by a central fixation cross presented for 150 ms. Then a symbol-string was displayed at the center of the screen for 500 ms, followed by a mask of four schematic snowflakes presented for 150 ms (Figure 1 C).

In the global report task, children had to report by mouse-clicking as many symbols as possible immediately after the string disappeared, by selecting symbols from the panel (Figure 1B). The score of the global report task was the number of accurately reported symbols (identity not location) across the 16 experimental trials with a maximum score equal to 64.

In the partial report task, 32 four-symbol strings were presented, followed by the mask, similar to the global report task. However, one of the snowflakes presented after the symbol-string was highlighted, and children were asked to select which of the two symbols presented below the snowflakes was previously shown in this position (Figure 1D). The experimental trials were preceded by 5 training trails. In the training trials participants were given feedback. No feedback was given in the experimental trials. The score of the partial report task was the percentage of accurately selected symbols across the 32 experimental trials.


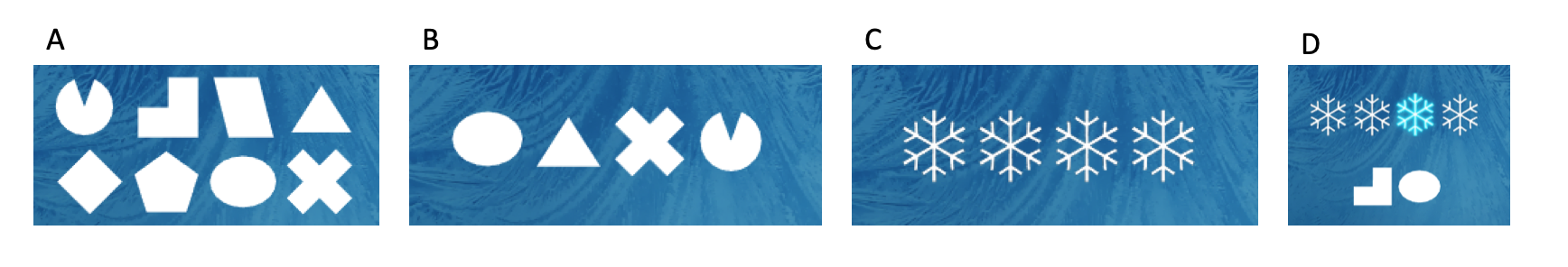


Figure S1. Symbols used in the visual attention span tasks.

### Implicit learning

In the computerized non-verbal implicit learning task, children were instructed to press buttons corresponding to the four holes arranged in a shape of cross on the screen. Each hole corresponds to the four buttons on the Cedrus keyboard. The animated story behind the task is “to catch” a gnome that appears in the hole by pressing a corresponding key. The design was similar to the serial reaction-time task in previous studies (Menghini et al., 2006; Vicari, 2005) but the same moving visual cue was used instead of a set of different colors. In the 8 trial sessions, children had to score at least 75% correct responses to start the experiment. The experimental sessions contained 6 blocks of 48 stimulus-response pairs in each. Between blocks, the picture of the clepsydra appeared on the screen for 3 seconds. The reaction time was measured after the gnome appeared on the screen. The next trial started 50 ms after a child pressed a button on the Cedrus keyboard (regardless of the accuracy). In the first and sixth block the gnome appeared in holes in a pseudo-random order, no more than two times in a row in the same hole. In blocks 2 to 5, the 8 stimulus-response pairs were repeated 6 times in a sequence (position: 42313241). Children were not informed about the sequence. However, the decrease of reaction time was expected as the child learned the sequence implicitly. The final score from the task that reflects the level of implicit learning (Vicari et al., 2003) was defined as a difference in reaction time medians between the last random block (6th block) and the last ordered block with a sequence (5th block).

# S2. Distributions of raw scores for all tasks

This supplementary material presents the descriptive statistics of the variables used in assessment of reading and cognitive skills (see the description of the tasks in Supplementary material S1). The raw data used for the calculations is available from https://osf.io/mj32v/

### Table S2.1. Mean, SD, minimal and maximal values for each of the raw variables used to calculate factors or used directly in the analyses (N = 211).

| Variable | Reliability | Comment | Mean | SD | Min | Max |
| --- | --- | --- | --- | --- | --- | --- |
| Single word reading  (sten score) | .93 - .96 ab | READING factor (accuracy) | 4.93 | 2.15 | 1.00 | 10.00 |
| Pseudoword reading  (sten score) | .93 - .94 ac | READING factor (fluency) | 4.31 | 1.85 | 1.00 | 9.00 |
| Reading with lexical decision (sten score) | .79 - .93 ac | READING factor (fluency) | 4.74 | 2.22 | 1.00 | 10.00 |
| Phonological awareness task (sten score) | .82 - .88 ab | PHONOLOGY factor | 4.38 | 1.94 | 1.00 | 10.00 |
| Phoneme deletion task  (sten score) | .51 - .71 ab | PHONOLOGY factor | 4.82 | 2.07 | 1.00 | 10.00 |
| RAN  (z-score) | .40 - .90 ac |  | 0.00 | 1.00 | -4.90 | 2.04 |
| SELECTIVE_ATTENTION (z-score) | .96 d |  | 0.00 | 1.00 | -3.71 | 2.75 |
| RHYTHM COMPARISON  (z-score) | .53 e |  | 0.00 | 1.00 | -3.97 | 2.24 |
| TONE COMPARISON  (z-score) | ND f |  | 0.00 | 1.00 | -6.01 | 2.66 |
| VAS global reporting  (raw score, 0 - 64) | ND f | VAS factor | 37.44 | 4.76 | 22.00 | 52.00 |
| VAS partial reporting  (percentage of correct) | .81 e | VAS factor | 57.08 | 10.53 | 31.25 | 81.25 |
| IMPLICIT LEARNING  (z-score) | .81 e |  | 0.00 | 1.00 | -5.15 | 2.24 |
| FHD |  |  | 0.43 | 0.50 | 0.00 | 1.00 |
| SES |  |  | 102.64 | 21.09 | 28.00 | 129.00 |
| Age |  |  | 10.06 | 1.10 | 6.91 | 12.49 |
| Nonverbal IQ |  |  | 115.70 | 13.15 | 89.00 | 149.00 |

*Notes:*

1. The reliability ratings for the standardized tests including in the battery used for dyslexia diagnosis are reported following the battery manual (Bogdanowicz et al., 2009)
2. Cronbach’s alpha is reported. The range depends on the sample (general sample from the norming study vs validation sample with dyslexia in the norming study) and test version (for younger and older children)
3. Pearson *r* is reported as a measure of test-retest reliability. The range depends on the sample (general sample from the norming study vs validation sample with dyslexia in the norming study) and test version (for younger and older children)
4. Spearman-Brown reliability reported following the manual for Polish adaptation of IDS (Jaworowska et al., 2012).
5. Split-half reliability (odd vs even items) is reported based on the sample included in the current study.
6. No data needed to calculate the reliability of the test is available.

# S3. Distribution of the deficits in children with dyslexia and in typical readers

This supplementary material presents the distribution of deficits in children (n = 51) with and in the control group (n = 71). The material is organized in four tables, either aggregated for deficits (Tables S3.1 and S3.3) or for individual participants (Tables S3.2 and S3.4).

The original data used for preparation of the material is available at https://osf.io/mj32v/

### Table S3.1. The distribution of the deficits in the group with dyslexia.

| Deficit | Children with a deficit  (-1 SD) | Children with a deficit  (-1.65 SD) |
| --- | --- | --- |
| *NONE* | D4M021 D5M011 T3M011 | D3F018 D3F024 D3M007 D3M029 D3M036 D4F013 D4F028 D4M021 D4M037 D5M011 D5M028 D5M038 T3M011 |
| PHONOLOGICAL | D4F027 D4F028 D5F015 D5M035 | D3F013 D3M005 D3M027 D4F010 D4F027 D4M002 D4M010 D4M014 D4M026 D5F015 D5F019 D5M030 D5M035 D5M040 |
| PHONOLOGICAL + IMPLICIT LEARNING | D4F015 D5M026 |  |
| PHONOLOGICAL + IMPLICIT LEARNING + TONE COMPARISON | D4M026 |  |
| PHONOLOGICAL + SELECTIVE ATTENTION + RHYTHM COMPARISON | D4M002 D5M040 |  |
| PHONOLOGICAL + SELECTIVE ATTENTION + TONE COMPARISON | D3F018 D5M045 | D5M045 |
| PHONOLOGICAL + RHYTHM COMPARISON | D4M037 | D3F002 |
| PHONOLOGICAL + VISUAL ATTENTION SPAN | D4F019 D5F019 D5M029 | D3F011 D4F019 D5M029 |
| PHONOLOGICAL + VISUAL ATTENTION SPAN + TONE COMPARISON | D3F007 |  |
| PHONOLOGICAL + VISUAL ATTENTION SPAN + TONE COMPARISON + RHYTHM COMPARISON | D3F002 |  |
| PHONOLOGICAL + RAN | D3F013 D3M024 D3M027 D3M035 D5M028 D5M030 | D3M010 D3M050 D4M030 T3M015 |
| PHONOLOGICAL + RAN + IMPLICIT LEARNING |  | D3F026 |
| PHONOLOGICAL + RAN + RHYTHM COMPARISON | D3F022 | D3F022 |
| PHONOLOGICAL + RAN + TONE COMPARISON | D3M052 | D3M052 |
| PHONOLOGICAL + RAN + SELECTIVE ATTENTION | D3M010 |  |
| PHONOLOGICAL + RAN + VISUAL ATTENTION SPAN | D3M050 D4M014 |  |
| PHONOLOGICAL + RAN + SELECTIVE ATTENTION + IMPLICIT LEARNING | D3F026 |  |
| PHONOLOGICAL + RAN + SELECTIVE ATTENTION + RHYTHM COMPARISON | D4F010 D4M030 |  |
| PHONOLOGICAL + RAN + VISUAL ATTENTION SPAN + RHYTHM COMPARISON | T3M015 |  |
| PHONOLOGICAL + RAN + VISUAL ATTENTION SPAN + TONE COMPARISON | D3F011 |  |
| PHONOLOGICAL + RAN + VISUAL ATTENTION SPAN + RHYTHM COMPARISON + SELECTIVE ATTENTION | D4M010 |  |
| PHONOLOGICAL + RAN + VISUAL ATTENTION SPAN + TONE COMPARISON + SELECTIVE ATTENTION | D3M005 |  |
| RAN | D5M016 D5M038 | D3M004 D3M024 D3M035 D5M016 |
| RAN + IMPLICIT LEARNING | D4M003 | D3M013 |
| RAN + SELECTIVE ATTENTION | D3M004 |  |
| RAN + TONE COMPARISON | D3M029 | T3M006 |
| RAN + VISUAL ATTENTION SPAN | D3M007 D4F013 |  |
| RAN + TONE COMPARISON + IMPLICIT LEARNING | D3M013 T3M006 |  |
| RAN + TONE COMPARISON + RHYTHM COMPARISON | D3F024 |  |
| IMPLICIT LEARNING | D4M016 | D3F027 D4F015 D4M003 D4M016 D5M026 |
| IMPLICIT LEARNING + RHYTHM COMPARISON | D3F027 |  |
| VISUAL ATTENTION SPAN | D3M036 | D3F007 |

###

### Table S3.2. The deficits of individual participants in the group with dyslexia.

| **Code** | **Deficits at -1 SD** | **Deficits at -1.65 SD** |
| --- | --- | --- |
| D3F002 | PHO, VAS, RHY, TON | PHO, RHY |
| D3F007 | PHO, VAS, TON | VAS |
| D3F011 | PHO, RAN, VAS, TON | PHO, VAS |
| D3F013 | PHO, RAN | PHO |
| D3F018 | PHO, SA, TON |  |
| D3F022 | PHO, RAN, RHY | PHO, RAN, RHY |
| D3F024 | RAN, RHY, TON |  |
| D3F026 | PHO, RAN, SA, IL | PHO, RAN, IL |
| D3F027 | IL, RHY | IL |
| D3M004 | RAN, SA | RAN |
| D3M005 | PHO, RAN, VAS, SA, TON | PHO |
| D3M007 | RAN, VAS |  |
| D3M010 | PHO, RAN, SA | PHO, RAN |
| D3M013 | RAN, IL, TON | RAN, IL |
| D3M024 | PHO, RAN | RAN |
| D3M027 | PHO, RAN | PHO |
| D3M029 | RAN, TON |  |
| D3M035 | PHO, RAN | RAN |
| D3M036 | VAS |  |
| D3M050 | PHO, RAN, VAS | PHO, RAN |
| D3M052 | PHO, RAN, TON | PHO, RAN, TON |
| D4F010 | PHO, RAN, SA, RHY | PHO |
| D4F013 | RAN, VAS |  |
| D4F015 | PHO, IL | IL |
| D4F019 | PHO, VAS | PHO, VAS |
| D4F027 | PHO | PHO |
| D4F028 | PHO |  |
| D4M002 | PHO, SA, RHY | PHO |
| D4M003 | RAN, IL | IL |
| D4M010 | PHO, RAN, VAS, SA, RHY | PHO |
| D4M014 | PHO, RAN, VAS | PHO |
| D4M016 | IL | IL |
| D4M021 |  |  |
| D4M026 | PHO, IL, TON | PHO |
| D4M030 | PHO, RAN, SA, RHY | PHO, RAN |
| D4M037 | PHO, RHY |  |
| D5F015 | PHO | PHO |
| D5F019 | PHO, VAS | PHO |
| D5M011 |  |  |
| D5M016 | RAN | RAN |
| D5M026 | PHO, IL | IL |
| D5M028 | PHO, RAN |  |
| D5M029 | PHO, VAS | PHO, VAS |
| D5M030 | PHO, RAN | PHO |
| D5M035 | PHO | PHO |
| D5M038 | RAN |  |
| D5M040 | PHO, SA, RHY | PHO |
| D5M045 | PHO, SA, TON | PHO, SA, TON |
| T3M006 | RAN, IL, TON | RAN, TON |
| T3M011 |  |  |
| T3M015 | PHO, RAN, VAS, RHY | PHO, RAN |

*Note:* PHO - phonological, RAN - rapid automatized naming, VAS - visual attention span, SA - selective attention, IL - implicit learning, RHY - rhythm comparison, TON - tone comparison

###

### Table S3.3. The distribution of the deficits in the control group.

| Deficit | Children with a deficit  (-1 SD) | Children with a deficit  (-1.65 SD) |
| --- | --- | --- |
| *NONE* | *D3F004 D3F010 D3M015 D4F002 D4F006 D4M022 D5F005 D5F009 D5F017 D5F020 D5M023 T3F002 T3F012 T3M001 T4F006 T4F010 T4M008 T4M011 T5F001 T5F003 T5F007 T5M005* | *D3F004 D3F005 D3F010 D3F016 D3M003 D3M014 D3M015 D3M018 D3M020 D3M037 D3M038 D3M041 D4F002 D4F004 D4F006 D4F008 D4F014 D4F023 D4M022 D4M029 D5F005 D5F007 D5F009 D5F014 D5F017 D5F020 D5F022 D5M006 D5M023 T3F002 T3F007 T3F012 T3F013 T3F014 T3M001 T3M004 T3M012 T4F006 T4F007 T4F008 T4F010 T4M007 T4M008 T4M009 T4M011 T5F001 T5F003 T5F005 T5F006 T5F007 T5M004 T5M005* |
| PHONOLOGICAL | D3M038 D5M002 T3F013 T4F008 T4M007 T5F005 | D5M002 |
| PHONOLOGICAL + IMPLICIT LEARNING | D3M047 |  |
| PHONOLOGICAL + SELECTIVE ATTENTION | T3F003 |  |
| PHONOLOGICAL + RHYTHM COMPARISON | D3M037 |  |
| PHONOLOGICAL + VISUAL ATTENTION SPAN | D3M020 |  |
| PHONOLOGICAL + VISUAL ATTENTION SPAN + SELECTIVE ATTENTION | D4F026 |  |
| PHONOLOGICAL + RAN + RHYTHM COMPARISON + TONE COMPARISON | T4M002 |  |
| RAN | D3M003 T5M001 | D3M021 T3F010 T5M001 |
| RAN + RHYTHM COMPARISON | D5F014 |  |
| RAN + SELECTIVE ATTENTION | D4F008 D4M013 T3F001 | D4M013 |
| RAN + TONE COMPARISON | D3M021 |  |
| RAN + VISUAL ATTENTION SPAN | T3F007 T3F010 |  |
| RAN + TONE COMPARISON + IMPLICIT LEARNING | T3M004 |  |
| IMPLICIT LEARNING | D3F005 D3M041 D4M038 T4F007 T5F006 T5M004 | D3M047 D4M038 T3M008 |
| IMPLICIT LEARNING + RHYTHM COMPARISON | T3M008 |  |
| IMPLICIT LEARNING + TONE COMPARISON | D3F016 |  |
| RHYTHM COMPARISON | D5F007 D5M006 D3M018 | T4M002 |
| RHYTHM COMPARISON + TONE COMPARISON | D4M015 |  |
| SELECTIVE ATTENTION |  | D4F026 T3F001 T3F003 |
| SELECTIVE ATTENTION + RHYTHM COMPARISON | D4F014 | D3M039 |
| SELECTIVE ATTENTION + IMPLICIT LEARNING + RHYTHM COMPARISON | D3M039 |  |
| SELECTIVE ATTENTION + IMPLICIT LEARNING + TONE COMPARISON | T5F002 | T5F002 |
| TONE COMPARISON | D3M014 D4F004 T4M012 | D4M015 T4M012 |
| VISUAL ATTENTION SPAN | D5F022 T3M012 T4M009 T5M002 | D4F007 T5M002 |
| VISUAL ATTENTION SPAN + IMPLICIT LEARNING | D4F007 T4M003 | T4M003 |
| VISUAL ATTENTION SPAN + RHYTHM COMPARISON | T3F014 |  |
| VISUAL ATTENTION SPAN + RHYTHM COMPARISON + TONE COMPARISON | D4M029 |  |
| VISUAL ATTENTION SPAN + SELECTIVE ATTENTION + IMPLICIT LEARNING | D4F023 |  |

### Table S3.4. The deficits of individual participants in the control group.

| **Code** | **Deficits at -1 SD** | **Deficits at -1.65 SD** |
| --- | --- | --- |
| D3F004 |  |  |
| D3F005 | IL |  |
| D3F010 |  |  |
| D3F016 | IL, TON |  |
| D3M003 | RAN |  |
| D3M014 | TON |  |
| D3M015 |  |  |
| D3M018 | RHY |  |
| D3M020 | PHO, VAS |  |
| D3M021 | RAN, TON | RAN |
| D3M037 | PHO, RHY |  |
| D3M038 | PHO |  |
| D3M039 | SA, IL, RHY | SA, RHY |
| D3M041 | IL |  |
| D3M047 | PHO, IL | IL |
| D4F002 |  |  |
| D4F004 | TON |  |
| D4F006 |  |  |
| D4F007 | VAS, IL | VAS |
| D4F008 | RAN, SA |  |
| D4F014 | SA, RHY |  |
| D4F023 | VAS, SA, IL |  |
| D4F026 | PHO, VAS, SA | SA |
| D4M013 | RAN, SA | RAN, SA |
| D4M015 | RHY, TON | TON |
| D4M022 |  |  |
| D4M029 | VAS, RHY, TON |  |
| D4M038 | IL | IL |
| D5F005 |  |  |
| D5F007 | RHY |  |
| D5F009 |  |  |
| D5F014 | RAN, RHY |  |
| D5F017 |  |  |
| D5F020 |  |  |
| D5F022 | VAS |  |
| D5M002 | PHO | PHO |
| D5M006 | RHY |  |
| D5M023 |  |  |
| T3F001 | RAN, SA | SA |
| T3F002 |  |  |
| T3F003 | PHO, SA | SA |
| T3F007 | RAN, VAS |  |
| T3F010 | RAN, VAS | RAN |
| T3F012 |  |  |
| T3F013 | PHO |  |
| T3F014 | VAS, RHY |  |
| T3M001 |  |  |
| T3M004 | RAN, IL, TON |  |
| T3M008 | IL, RHY | IL |
| T3M012 | VAS |  |
| T4F006 |  |  |
| T4F007 | IL |  |
| T4F008 | PHO |  |
| T4F010 |  |  |
| T4M002 | PHO, RAN, RHY, TON | RHY |
| T4M003 | VAS, IL | VAS, IL |
| T4M007 | PHO |  |
| T4M008 |  |  |
| T4M009 | VAS |  |
| T4M011 |  |  |
| T4M012 | TON | TON |
| T5F001 |  |  |
| T5F002 | SA, IL, TON | SA, IL, TON |
| T5F003 |  |  |
| T5F005 | PHO |  |
| T5F006 | IL |  |
| T5F007 |  |  |
| T5M001 | RAN | RAN |
| T5M002 | VAS | VAS |
| T5M004 | IL |  |
| T5M005 |  |  |

*Note:* PHO - phonological, RAN - rapid automatized naming, VAS - visual attention span, SA - selective attention, IL - implicit learning, RHY - rhythm comparison, TON - tone comparison

# S4. Effects of cognitive factors on reading accuracy and fluency

In the main analyses we treated reading level as a single factor constructed from three different tasks measuring reading accuracy and fluency. Here, we tested the effects of cognitive factors on accuracy and fluency separately. The reading accuracy was measured using a single task, the reading fluency was measured using two tasks which were averaged for this analysis (correlation between tasks 0.72). We repeated the regression analysis measuring the impact of various cognitive factors on reading abilities in the whole studied population. Tables S4.1 and S4.2 present the results for reading accuracy and fluency, respectively. In both of these analyses only PHONOLOGY and RAN were significant predictors of reading abilities. Then, we performed the analysis of the effect of RAN and PHONOLOGY on reading accuracy and fluency across different levels of the reading ability. We used the same procedure as described in the main manuscript for the whole reading factor (Figure 3 in the main manuscript). Figures S4.1 and S4.2 present results of these analyses. They are qualitatively similar and display the same pattern: phonology coefficients are low in the poor readers group.

### Table S4.1. Coefficients of the linear regression predicting reading accuracy.

|  | Beta | 95% CI^1^ | *p*-value |
| --- | --- | --- | --- |
| PHONOLOGY | 0.46 | 0.32, 0.59 | <0.001 |
| RAN | 0.22 | 0.08, 0.35 | 0.002 |
| TONE COMPARISON | 0.04 | -0.09, 0.16 | 0.571 |
| VAS | 0.03 | -0.10, 0.15 | 0.668 |
| SELECTIVE ATTENTION | -0.09 | -0.23, 0.05 | 0.198 |
| RHYTHM COMPARISON | 0.03 | -0.10, 0.16 | 0.651 |
| IMPLICIT LEARNING | 0.01 | -0.11, 0.13 | 0.872 |
| FHD | -0.03 | -0.15, 0.10 | 0.688 |
| SES | -0.06 | -0.19, 0.06 | 0.327 |
| Age | -0.13 | -0.27, 0.02 | 0.091 |
| Nonverbal IQ | 0.03 | -0.10, 0.16 | 0.681 |
| ^1^CI = Confidence Interval | | | |

### Table S4.2. Coefficients of the linear regression predicting reading fluency.

|  | Beta | 95% CI^1^ | p-value |
| --- | --- | --- | --- |
| PHONOLOGY | 0.36 | 0.23, 0.50 | <0.001 |
| RAN | 0.31 | 0.17, 0.44 | <0.001 |
| TONE COMPARISON | 0.12 | -0.01, 0.25 | 0.074 |
| VAS | -0.10 | -0.22, 0.03 | 0.148 |
| SELECTIVE ATTENTION | -0.05 | -0.20, 0.09 | 0.465 |
| RHYTHM COMPARISON | -0.07 | -0.20, 0.06 | 0.295 |
| IMPLICIT LEARNING | 0.07 | -0.05, 0.20 | 0.258 |
| FHD | -0.07 | -0.20, 0.05 | 0.254 |
| SES | 0.00 | -0.13, 0.13 | 0.975 |
| Age | -0.08 | -0.23, 0.07 | 0.288 |
| Nonverbal IQ | 0.00 | -0.14, 0.13 | 0.961 |
| ^1^CI = Confidence Interval | | | |

Figure S4.1. Sensitivity analysis of the regression coefficients as a function of the data sample. Linear regression model predicted Reading Accuracy level based on Phonology and RAN (with age, SES, FHD, and nonverbal IQ controlled for). The same model was fitted repeatedly using data samples of different sizes (sample size marked on the x-axis), standardized coefficients of Phonology and RAN were extracted from the fitted model (standardized coefficients marked on the y-axis). Samples were formed as subsets of the original sample increasing in size, while data points were sorted by Reading values a) in ascending order, b) in descending order. If the value of the standardized coefficient is similar for all sample sizes, it means that the predictor is equally good across the full range of Reading Accuracy values.


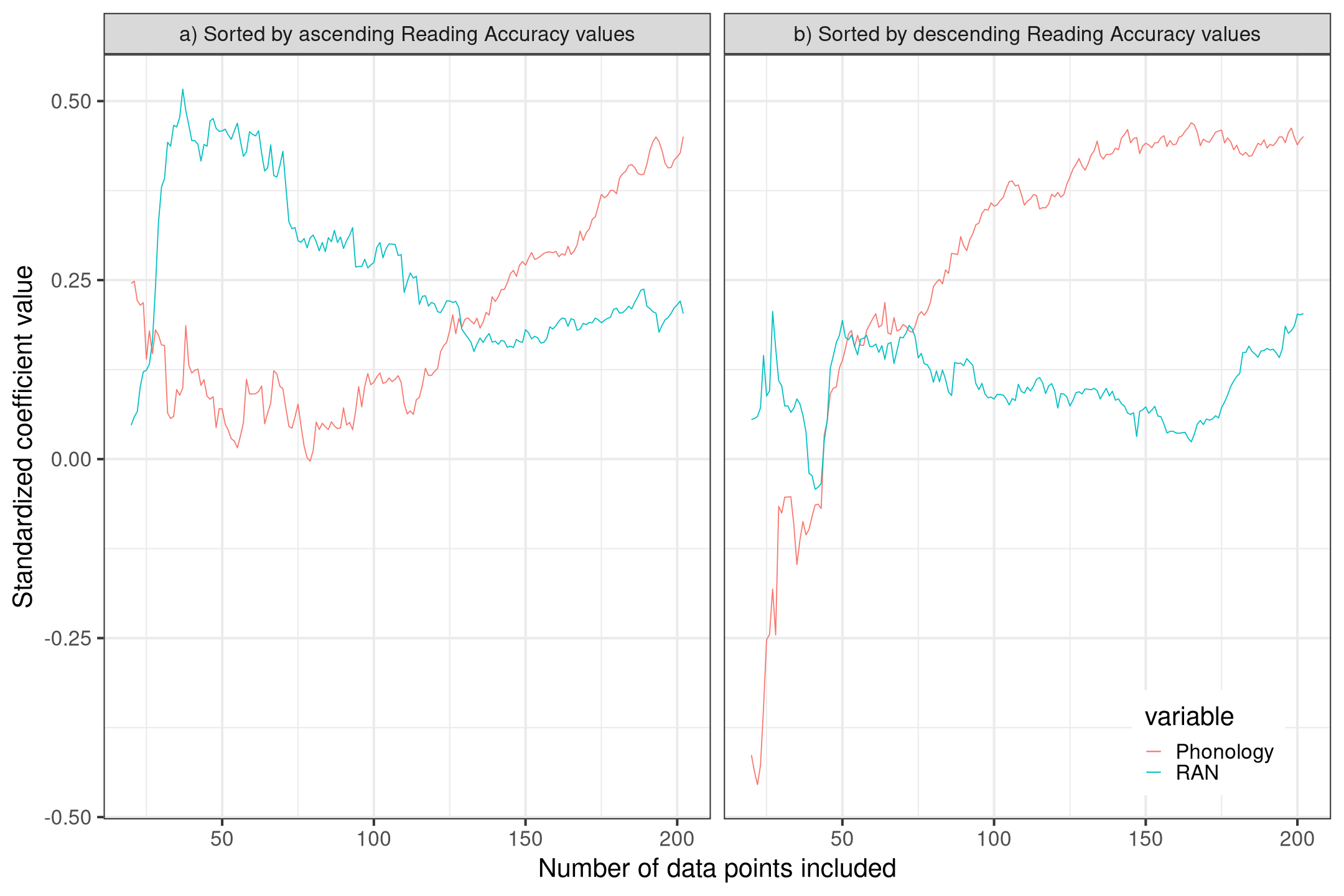


Figure S4.2. Sensitivity analysis of the regression coefficients as a function of the data sample. Linear regression model predicted Reading Fluency level based on Phonology and RAN (with age, SES, FHD, and nonverbal IQ controlled for). The same model was fitted repeatedly using data samples of different sizes (sample size marked on the x-axis), standardized coefficients of Phonology and RAN were extracted from the fitted model (standardized coefficients marked on the y-axis). Samples were formed as subsets of the original sample increasing in size, while data points were sorted by Reading values a) in ascending order, b) in descending order. If the value of the standardized coefficient is similar for all sample sizes, it means that the predictor is equally good across the full range of Reading Fluency values.


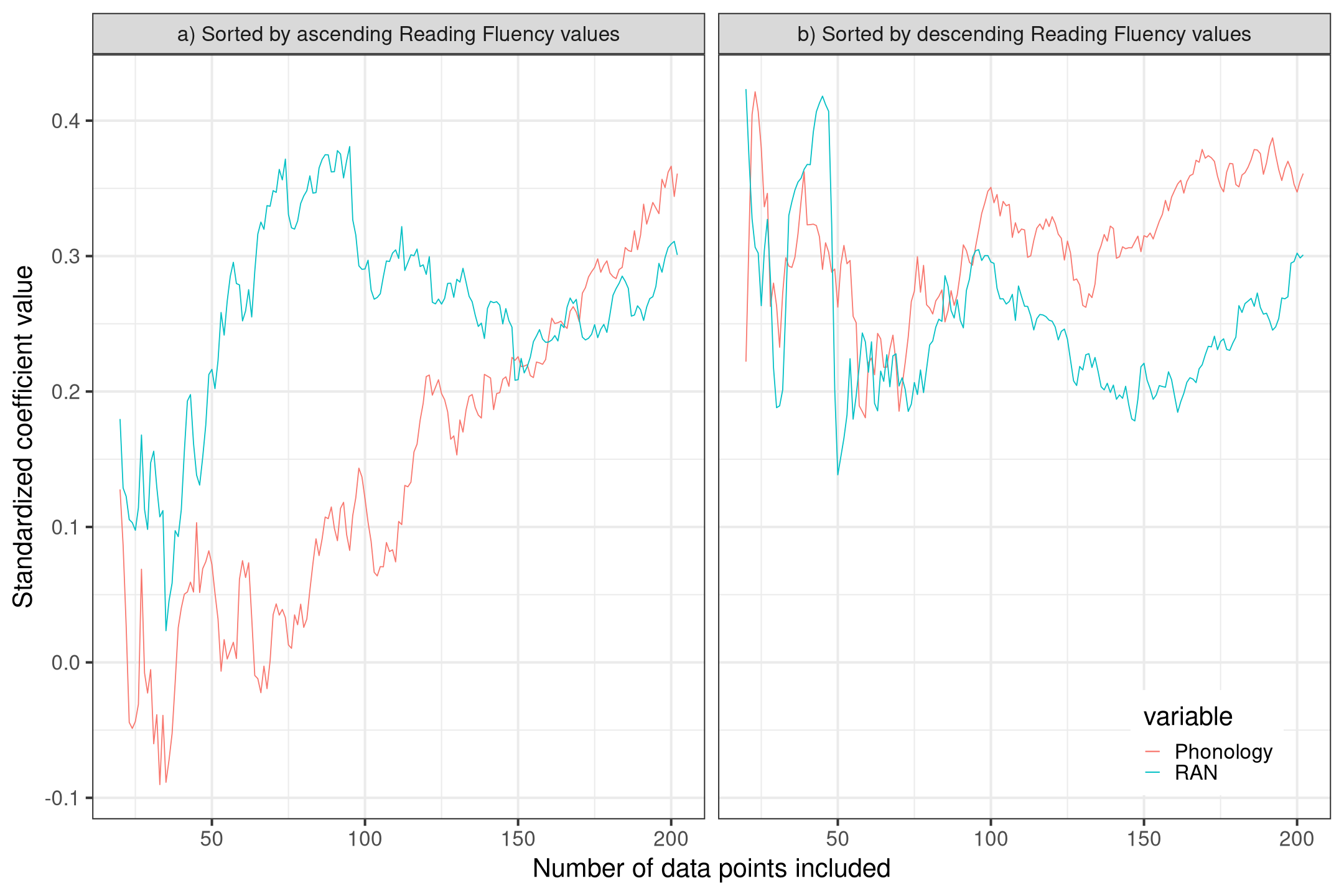

Supplement: Supplementary file 1 — Supporting information [file DESC-25-0-s001.docx]
